# Mortality estimates by age and sex among persons living with HIV after ART initiation in Zambia using electronic medical records supplemented with tracing a sample of lost patients: A cohort study

**DOI:** 10.1371/journal.pmed.1003107

**Published:** 2020-05-13

**Authors:** Andrew D. Kerkhoff, Kombatende Sikombe, Ingrid Eshun-Wilson, Izukanji Sikazwe, David V. Glidden, Jake M. Pry, Paul Somwe, Laura K. Beres, Sandra Simbeza, Chanda Mwamba, Chama Bukankala, Cardinal Hantuba, Carolyn Bolton Moore, Charles B. Holmes, Nancy Padian, Elvin H. Geng

**Affiliations:** 1 Division of HIV, Infectious Diseases and Global Medicine, Zuckerberg San Francisco General Hospital and Trauma Center, University of California, San Francisco, San Francisco, California, United States of America; 2 Centre for Infectious Disease Research in Zambia, Lusaka, Zambia; 3 Division of Infectious Diseases, Department of Medicine, Washington University, St. Louis, Missouri, United States of America; 4 Center for Dissemination and Implementation, Institute for Public Health, Washington University, St. Louis, Missouri, United States of America; 5 Johns Hopkins University, Baltimore, Maryland, United States of America; 6 University of Alabama at Birmingham, Birmingham, Alabama, United States of America; 7 Georgetown University, Washington, District of Columbia, United States of America; 8 University of California, Berkeley, Berkeley, California, United States of America; University of Bern, SWITZERLAND

## Abstract

**Background:**

Men in sub-Saharan Africa have lower engagement and retention in HIV services compared to women, which may result in differential survival. However, the true magnitude of difference in HIV-related mortality between men and women receiving antiretroviral therapy (ART) is incompletely characterized.

**Methods and findings:**

We evaluated HIV-positive adults ≥18 years old newly initiating ART in 4 Zambian provinces (Eastern, Lusaka, Southern, and Western). In addition to mortality data obtained from routine electronic medical records, we intensively traced a random sample of patients lost to follow-up (LTFU) and incorporated tracing outcomes through inverse probability weights. Sex-specific mortality rates and rate differences were determined using Poisson regression. Parametric g-computation was used to estimate adjusted mortality rates by sex and age. The study included 49,129 adults newly initiated on ART between August 2013 and July 2015; overall, the median age among patients was 35 years, the median baseline CD4 count was 262 cells/μl, and 37.2% were men. Men comprised a smaller proportion of individuals starting ART (37.2% versus 62.8%), tended to be older (median age 37 versus 33 years), and tended to have lower CD4 counts (median 220 versus 289 cells/μl) at the time of ART initiation compared to women. The overall rate of mortality among men was 10.3 (95% CI 8.2–12.4) deaths/100 person-years (PYs), compared to 5.5 (95% CI 4.3–6.8) deaths/100 PYs among women (difference +4.7 [95% CI 2.3–7.2] deaths/100 PYs; *p <* 0.001). Compared to women in the same age groups, men’s mortality rates were particularly elevated among those <30 years old (+6.7 deaths/100 PYs difference), those attending rural health centers (+9.4 deaths/100 PYs difference), those who had an initial CD4 count < 100 cells/μl (+9.2 deaths/100 PYs difference), and those who were unmarried (+8.0 deaths/100 PYs difference). After adjustment for potential confounders and mediators including CD4 count, a substantially higher mortality rate was predicted among men <30 years old compared to women of the same age, while women ≥50 years old had a mortality rate similar to that of age-matched men, but considerably higher than that predicted among young women (<30 years old). No clinically significant differences were evident with respect to rates of facility transfer or care disengagement between men and women. The main study limitations were the inability to successfully ascertain outcomes in all patients selected for tracing and missing clinical and laboratory data due to the use of medical records.

**Conclusions:**

In this study, we found that among HIV-positive adults newly initiating ART, mortality among men exceeded mortality among women; disparities were most pronounced among young patients. Older women, however, also experienced high mortality. Specific interventions for men and older women at highest mortality risk are needed to improve HIV treatment outcomes.

## Abstract 2

In a cohort study, Andrew Kerkhoff and colleagues use a sampling approach to identify patients lost to follow-up after starting antiretroviral treatment and investigate mortality rates across demographic groups.

## Author summary

**Why was this study done?:**

- Despite many studies demonstrating differences in HIV-related outcomes between men and women on antiretroviral therapy (ART) in sub-Saharan Africa, few studies use a probability sample that would enable them to offer regionally representative estimates.
- Many studies taken from routine service delivery settings are unable to account for outcomes among individuals lost to follow-up, which may threaten the validity of estimates comparing mortality in men and women.
- Furthermore, whether differences in survival between men and women vary across other important sociodemographic characteristics (such as age) remains underexplored.

**What did the researchers do and find?:**

- We used a multistage sampling approach to enumerate an analysis population of HIV-positive patients visiting public health facilities in 4 provinces in Zambia (Lusaka, Southern, Eastern, and Western).
- We estimated the association between sex and mortality overall and by age, adjusting for other sociodemographic and clinical characteristics.
- Of 49,129 adults newly initiating ART, the mortality rate was almost twice as high in men compared to women.
- Analysis of age-by-sex interactions revealed particularly elevated mortality among young males (as compared to females of the same age). While mortality rates appeared to fall with age among men, mortality rates rose with age among women, and by 50 years of age, women had a 2–3 times higher rate of death compared to women under 30.

**What do these findings mean?:**

- Among adults living with HIV in Zambia, men on average experience greater mortality compared to women, but this difference varies markedly by age, even after adjustment for other sociodemographic and clinical characteristics (e.g., baseline level of immunosuppression).
- Additional means of engaging and supporting younger men in HIV care is urgently needed and may include improved access to self-testing, use of financial incentives, and male-friendly services that feature flexible hours, an integrated multi-disease care model, and reduced visit frequency.
- Rising mortality associated with age in women greater than would be expected in the general population suggests that health services targeting women of reproductive age may be in part responsible for good clinical outcomes in younger women, but also highlights the need for specific programs to engage older women in care.

## Introduction

Men in sub-Saharan Africa have poorer engagement at each step of the HIV care cascade, including lower rates of testing, antiretroviral therapy (ART) initiation, ART adherence, and program retention [1–6]. Men’s underrepresentation in HIV testing and treatment services has been dubbed “the blind spot” in the global HIV epidemic response [2,7]. This in large part is underpinned by differential health-seeking behavior among men compared to women, which in many settings may be heavily influenced by local gender norms and health service structures designed to engage women of reproductive age [8–11]. For many women, due to recommendations for preventing mother-to-child HIV transmission [12], the pregnancy or postpartum period is often the time when they are diagnosed with HIV, in antenatal or under-5 clinics, and initiated on ART [13], thus providing women with increased access to HIV care [4]. This aside, poor health-seeking behavior and engagement in HIV services among men is likely multifactorial and context-specific [8]. In many settings, men may perceive health facilities as spaces for women where they are likely to experience disrespect and lack of confidentiality [14,15]. HIV services with limited hours of operation, long wait times, or far physical distances may be worse for men because, in many settings, a man’s masculinity may be defined by his ability to provide for himself and his family, and therefore these structural factors may act as stronger barriers to men [1,8,16,17].

Despite well-described differential engagement in HIV care and differences in HIV-related outcomes between men and women, to date there have been only a few relatively unbiased estimates of sex-specific mortality in sub-Saharan Africa, most of which have been limited to South Africa [18–22]. In addition, few previous studies have taken a formal probability sample of a large geographical region that would enable them to offer regionally representative estimates, as this study seeks to do [23,24]. This study builds on previous work to provide a clearer picture of the public health realities around disparities (or potential lack thereof) between men and women in sub-Saharan Africa with respect to HIV care [4,25]. Furthermore, while several studies have shown higher mortality risk among men on ART [18–21,23,26], it is not well known if this differential mortality risk is consistent across all demographic groups and clinical settings, or whether mortality is “concentrated” among key subgroups such as younger adults or those who are unmarried; it is also not well known whether specific subgroups of women may be particularly vulnerable and experience higher rates of mortality. Therefore, to support improved individual survival among people living with HIV and progress towards global elimination goals [27,28], there is an urgent need to accurately characterize the risk of mortality among men and women receiving ART, and to better understand if mortality is disproportionally concentrated in certain subgroups.

Previously, we reported the results of a large multistage sampling survey among HIV patients in Zambia lost to follow-up (LTFU) from public ART services and intensively traced in order to supplement routine program data to derive unbiased estimates of care engagement and HIV-related mortality [22]. Before program data were weighted by outcomes from patients who were LTFU, “naïve” estimates significantly underestimated the proportion of individuals retained in care, and the proportion of patients who were viremic or died [22,29]. Notably, using program data alone, being male was a strong independent predictor of both viremia and mortality. We therefore undertook a similar analysis among adults living with HIV in Zambia who newly initiated ART to accurately assess differences in mortality between men and women, and across key subgroups. Our objectives were the following: (1) to determine sex-specific estimates of mortality among LTFU patients who were traced, (2) to determine “revised” sex-specific mortality rates among all patients after incorporating tracing outcomes, (3) to determine “revised” sex-specific mortality rates among key subgroups, including by age, after incorporating tracing outcomes, and (d) to determine “revised” sex-specific estimates of true HIV care status after incorporating tracing outcomes.

## Methods

### Setting and patient sampling

This study represents a prespecified subanalysis and is based on the findings of a previously reported parent study that sought to determine true mortality estimates among HIV-infected adults receiving ART in Zambia and that included the intention to examine interactions between sex and other factors on survival (see S1 Appendix for full analysis protocol) [22]. The sampling frame consisted of HIV-positive individuals 18 years or older seeking HIV care and treatment during a 2-year period from August 1, 2013, to July 31, 2015, at 1 of 64 public health facilities in 4 provinces (Eastern, Lusaka, Southern, and Western). As described previously, multistage sampling was used to enumerate an analysis population regionally representative of all HIV-positive patients receiving ART in 4 Zambian provinces; this tracing sampling included 32 facilities that were selected to allow for comparisons across 3 facility types (hospitals, urban health centers, and rural health centers) and the 4 provinces [22]. At each of the 32 sampled clinics, all patients LTFU—defined as being ≥90 days late for their last visit and not known to have transferred to a different facility or to have died—were identified. From these, a random sample of patients from each facility were selected for tracing [22]. Written informed consent was obtained from all patients traced in person; the consent procedures are detailed in the study protocol (S1 Appendix). The present analysis was restricted to those who newly initiated ART during the 2-year sampling period and who had at least 1 clinical encounter, in order to better understand care engagement patterns among men and women during early ART.

### Ethics

The University of Zambia Biomedical Research Ethics Committee (004-06-14) and the institutional review board of the University of Alabama, Birmingham School of Medicine (F160122006) approved both the protocol and the study. This study conforms to the STROBE statement for good practice in the reporting of cohort studies (S1 STROBE Checklist) [30].

### Procedures and measurements

The SmartCare electronic medical record system managed by the Zambian Ministry of Health was used to extract all data related to patients’ sociodemographic and clinical characteristics, as well as dates related to patient visits and individual outcomes. Among LTFU patients selected for intensive tracing between October 2015 and June 2016, care engagement and vital status were obtained using a combination of paper and electronic chart review, phone calls, and in-person visits via peer health workers [22]. A minimum of 3 tracing attempts were made for sampled patients. If patients were found to have died, the date, location, and cause of death were obtained from family or close contacts whenever possible. A patient was classified as alive if either the patient was contacted directly or a close informant was reached and reported that the patient was alive. When LTFU patients were reached through an in-person visit, they were asked whether they had seen a medical professional for HIV monitoring or treatment since last seen at their original clinic. A patient was determined to be retained in care (either at the original clinic or via transfer to a new clinic) only when this was confirmed by chart review or through self-report of patients who were contacted in person.

### Analysis

All estimates of care engagement status and vital status using tracing outcomes were determined through sampling weights that were inverse to the probability of selection at both the individual and facility level to yield regionally representative estimates (S1 Fig) [31,32].

After incorporating outcomes among those LTFU through probability weights, we first estimated differences in the rate of mortality between men and women overall and in subgroups defined by sociodemographic and clinical characteristics that were unadjusted for other patient characteristics. We used a forest plot to depict the range of associations between sex and mortality across demographic and clinical subgroups. Because barriers and facilitators to HIV care engagement and retention as well as co-morbidities and outcomes may differ substantially in men and women of different ages [2,33,34], we hypothesized that the strength of association between sex and mortality may vary by age. To assess the interaction between age and sex, we estimated adjusted mortality rates for men and women by age, with age treated as a continuous variable. We used parametric g-computation [35], a general technique to estimate causal effects from observational data, on a multivariable Poisson regression model to determine adjusted predicted mortality rates by sex and age (coded as a continuous variable), thus accounting for age as an effect modifier of the association between sex and mortality. Estimates were adjusted for the following characteristics at ART initiation: CD4 count, WHO stage, year, relationship status, education status, and province. The direct effect of sex on predicted mortality by age (adjusted rates of mortality) were visually represented using a plot.

The Aalen–Johansen method [36] was used to estimate the cumulative proportion of patients’ ART engagement status during the first 18 months of ART as defined by 1 of 4 mutually exclusive care states: (1) alive and in care at the original clinic, (2) alive and transferred to a new clinic (i.e., including both official and silent transfers), (3) alive but out of care, or (4) dead. The maximum duration of follow-up time that could be accrued was 2 years, and while some patients accrued 2 years of follow-up time, the relative proportion of such patients was small (*n* = 4,640; 9.4%); thus, analyses estimating the prevalence of clinical care states were censored after 18 months of ART to maximize observation time and control for the duration on ART, but minimize the loss of precision around estimates, especially for subgroup analyses. These results are presented graphically using mosaic plots among men and women overall, as well as stratified according to age category.

We had 2 types of missing data that we accounted for—missing predictor data (e.g., absence of baseline CD4 count; <20% missing) and missing vital status outcome. For the missing predictor data, we assumed “missingness at random,” which holds that after accounting for all measured covariates, missingness is not dependent on the value of the variable [37]. A regression model using sociodemographic and clinical predictors of successful vital status ascertainment was used to generate a predicted probability of ascertainment for each person sought, and we based our inverse probability weights on this (S1 Fig) [38]. All statistical analyses were conducted using Stata version 14.0.

## Results

### Baseline characteristics

Overall, 49,129 (29.7%) of 165,464 individuals newly initiated ART at 1 of 64 health facilities between August 1, 2013, and July 31, 2015, and were included in this analysis (S2 Fig). Men accounted for 37.2% (*n* = 18,271) of all individuals initiating ART. Compared to women, men tended to be older, have more advanced immunodeficiency, and be classified as having WHO stage III/IV disease at the time of ART initiation (Table 1). Men were also much more likely to be married compared to women, who were more likely to report being widowed (Table 1). Furthermore, at the time of ART initiation, men were more likely to have known tuberculosis (TB) disease and be on anti-TB therapy. We observed no differences in province of enrollment and type of facility between men and women.

**Table 1:** Baseline characteristics among people living with HIV newly initiating ART in 4 Zambian provinces between 2013 and 2015 stratified according to sex and age (in years) at ART initiation (*n* = 49,129).

	Overall (*n* = 49,129)	Males (*n* = 18,271)	Females (*n* = 30,858)	Males <30 years (*n* = 3,209)	Males 30–49.9 years (*n* = 12,969)	Males ≥50 years (*n* = 2,093)	Females <30 years (*n* = 11,555)	Females 30–49.9 years (*n* = 16,971)	Females ≥50 years (*n* = 2,332)
**Age at ART initiation (years), median (IQR)**	35 (29–41)	37 (32–44)	33 (27–40)	26 (24–28)	37 (33–41)	54 (51–59)	24 (22–27)	35 (32–40)	54 (51–59)
**CD4 count at ART initiation, median (IQR)**	262 (138–388)	220 (111–431)	289 (160–415)	257 (143–359)	211 (107–339)	210 (106–331)	316 (189–445)	274 (146–399)	256 (140–383)
<100	6,396 (13.0)	3,110 (17.0)	3,286 (10.7)	428 (13.3)	2,313 (17.8)	369 (17.6)	979 (8.5)	2,015 (11.9)	292 (12.5)
100–199	7,176 (14.6)	3,238 (17.7)	3,938 (12.8)	504 (15.7)	2,354 (18.2)	380 (18.2)	1,202 (10.4)	2,376 (14.0)	360 (15.4)
≥200	22,874 (46.6)	7,598 (41.6)	15,276 (49.5)	1,550 (48.3)	5,234 (40.4)	814 (38.9)	5,989 (51.8)	8,188 (48.3)	1,099 (47.1)
Unknown	12,683 (25.8)	4,325 (23.7)	8,538 (27.1)	727 (22.7)	3,068 (23.7)	530 (25.3)	3,385 (29.3)	4,392 (25.9)	581 (24.9)
**WHO stage at ART initiation**									
Stage I	23,149 (47.1)	7,217 (39.5)	15,932 (51.6)	1,425 (44.4)	5,215 (40.2)	770 (36.8)	6,613 (57.2)	8,533 (50.3)	988 (42.4)
Stage II	8,405 (17.1)	3,327 (18.2)	5,078 (16.5)	557 (17.4)	2,412 (18.6)	447 (21.4)	1,608 (13.9)	3,061 (18.0)	489 (21.0)
Stage III	9,343 (19.0)	4,711 (25.8)	4,632 (15.0)	715 (22.3)	3,228 (24.9)	486 (23.2)	1,314 (11.4)	2,567 (15.1)	469 (20.1)
Stage IV	777 (1.6)	389 (2.1)	388 (1.3)	71 (2.2)	287 (2.2)	31 (1.5)	110 (1.0)	228 (1.3)	50 (2.1)
Unknown	7,455 (15.2)	2,627 (14.4)	4,828 (15.7)	441 (13.7)	1,827 (14.1)	359 (17.2)	1,910 (16.5)	2,582 (15.2)	336 (14.4)
**TB status at ART initiation**									
Known prevalent TB	1,893 (3.9)	1,121 (6.1)	772 (2.5)	204 (6.4)	790 (6.1)	126 (6.1)	220 (1.9)	457 (2.7)	93 (4.0)
Unknown or negative for TB	47,236 (96.2)	17,150 (93.9)	30,086 (97.5)	3,005 (93.6)	12,179 (93.9)	1,948 (93.9)	11,335 (98.1)	16,514 (97.3)	2,211 (96.0)
**Province**									
Eastern	9,234 (18.8)	3,619 (19.8)	5,615 (18.2)	548 (17.1)	2,530 (19.5)	541 (25.8)	1,639 (14.2)	3,371 (19.9)	605 (25.9)
Lusaka	25,644 (52.2)	9,472 (51.8)	16,172 (52.4)	1,741 (54.3)	6,823 (52.6)	908 (43.4)	6,382 (55.2)	8,820 (52.0)	970 (41.6)
Southern	6,564 (13.4)	2,442 (13.4)	4,122 (13.4)	427 (13.3)	1,693 (13.0)	322 (15.4)	1,510 (13.1)	2,251 (13.3)	361 (15.5)
Western	7,687 (15.7)	2,738 (15.0)	4,949 (16.0)	493 (15.4)	1,923 (14.8)	322 (15.4)	2,024 (17.5)	2,529 (14.9)	396 (17.0)
**Facility type**									
Rural health center	5,442 (11.1)	1,923 (10.5)	3,519 (11.4)	288 (9.0)	1,325 (10.2)	310 (14.8)	1,199 (10.4)	1,971 (11.6)	349 (15.0)
Urban health center	28,053 (57.1)	10,368 (56.7)	17,685 (57.3)	1,917 (59.7)	7,473 (57.6)	978 (46.7)	7,015 (60.7)	9,556 (56.3)	1,114 (47.8)
Hospital	15,634 (31.8)	5,980 (32.7)	9,654 (31.3)	1,004 (31.3)	4,171 (32.2)	805 (38.5)	3,341 (28.9)	5,444 (32.1)	869 (37.3)
**Time from program entry to ART initiation (days), median (IQR)**	30 (14–167)	29 (14–113)	32 (14–212)	28 (14–79)	29 (14–119)	28 (14–148)	28 (11–147)	35 (14–265)	34 (14–259)
**Duration of ART (days), median (IQR)**	225 (63–407)	216 (58–408)	231 (70–407)	189 (46–388)	220 (59–413)	222 (59–410)	210 (57–380)	246 (78–426)	230 (69–414)
**Disclosure of HIV status to family or a friend at ART initiation**									
Yes	43,329 (88.2)	16,254 (89.0)	27,075 (87.7)	94 (2.9)	280 (2.2)	72 (3.4)	326 (2.8)	458 (2.7)	63 (2.7)
No	1,293 (2.6)	446 (2.4)	847 (2.7)	2,830 (88.2)	11,602 (89.5)	1,822 (87.1)	10,123 (87.6)	14,894 (87.8)	2,058 (88.3)
Unknown	4,507 (9.2)	1,571 (8.6)	2,936 (9.5)	285 (8.9)	1,087 (8.4)	199 (9.5)	1,106 (9.6)	1,619 (9.5)	211 (9.0)
**Highest education level**									
None	3,100 (6.3)	891 (4.9)	2,209 (7.2)	108 (3.4)	634 (4.9)	149 (7.1)	540 (4.7)	1,291 (7.6)	378 (16.2)
Lower/mid-basic	14,462 (29.4)	4,846 (26.5)	9,616 (31.2)	682 (21.3)	3,522 (27.2)	642 (30.7)	2,870 (24.8)	5,875 (34.6)	871 (16.2)
Upper basic/secondary	19,894 (40.5)	8,288 (45.4)	11,606 (37.6)	1,729 (53.9)	5,790 (44.6)	769 (36.7)	5,320 (46.0)	5,779 (34.1)	507 (21.7)
College/university	1,831 (3.7)	904 (4.9)	927 (3.0)	122 (3.8)	641 (4.9)	141 (6.7)	339 (2.9)	543 (3.2)	45 (1.9)
Unknown	9,842 (20.0)	3,342 (18.3)	6,500 (21.1)	568 (17.7)	2,382 (18.4)	392 (18.7)	2,486 (21.5)	3,483 (20.5)	531 (22.8)
**Relationship status**									
Unmarried	5,059 (10.3)	1,800 (9.9)	3,259 (10.6)	942 (29.4)	831 (6.4)	27 (1.3)	2,238 (19.4)	970 (5.7)	51 (2.2)
Married	25,988 (52.9)	10,954 (60.0)	15,034 (48.7)	1,375 (42.8)	8,197 (63.2)	1,382 (66.0)	5,715 (49.6)	8,671 (51.1)	648 (27.8)
Divorced	5,227 (10.6)	1,608 (8.8)	3,619 (11.7)	217 (6.8)	1,261 (9.7)	130 (6.2)	1,014 (8.8)	2,290 (13.5)	315 (13.5)
Widowed	3,397 (6.9)	642 (3.5)	2,755 (8.9)	32 (1.0)	450 (3.5)	160 (7.6)	278 (2.4)	1,752 (10.3)	725 (31.1)
Unknown	9,458 (19.3)	3,267 (17.9)	6,191 (20.1)	643 (20.0)	2,230 (17.2)	394 (18.8)	2,310 (20.0)	3,288 (19.4)	593 (25.4)

ART, antiretroviral therapy; CD4, cluster of differentiation 4; IQR, interquartile range; TB, tuberculosis; WHO, World Health Organization.

Stratification of baseline characteristics by age and sex revealed few differences across the joint strata with respect to the proportion with a CD4 cell count < 200 cells/μl or WHO stage III/IV disease (Table 1). However, women ≥50 years old had more advanced immunodeficiency than women <30 years (27.9% versus 18.9%) as well as a greater prevalence of WHO stage III/IV disease (22.2% versus 12.4%) compared to women <30 years. Patterns of relationship status were generally similar between men and women <50 years old; however, among men ≥50 years old, 66.0% were married compared to only 27.8% of women of the same age—comparatively large proportions of women ≥50 years old were either divorced (13.5%) or widowed (31.1%).

### Tracing outcomes among men and women LTFU

Among the 49,129 patients newly initiating ART, 10,217 (20.8%) were identified as LTFU. Men were slightly more likely than women to be classified as LTFU (21.8% versus 20.2%) (S1 Table). Among, those LTFU, approximately 10% (*n* = 993) were randomly traced, and updated clinical outcomes were obtained in 73.9% (*n* = 734) of these individuals (S1 Fig). Men ≥30 years appeared slightly more likely to be successfully traced than men <30 years, but this difference was not statistically significant (S1 Table). Baseline sociodemographic characteristics did not differ between those successfully traced and those who could not be traced (S2 Table).

### Mortality rates among men and women

**Fig 1:**
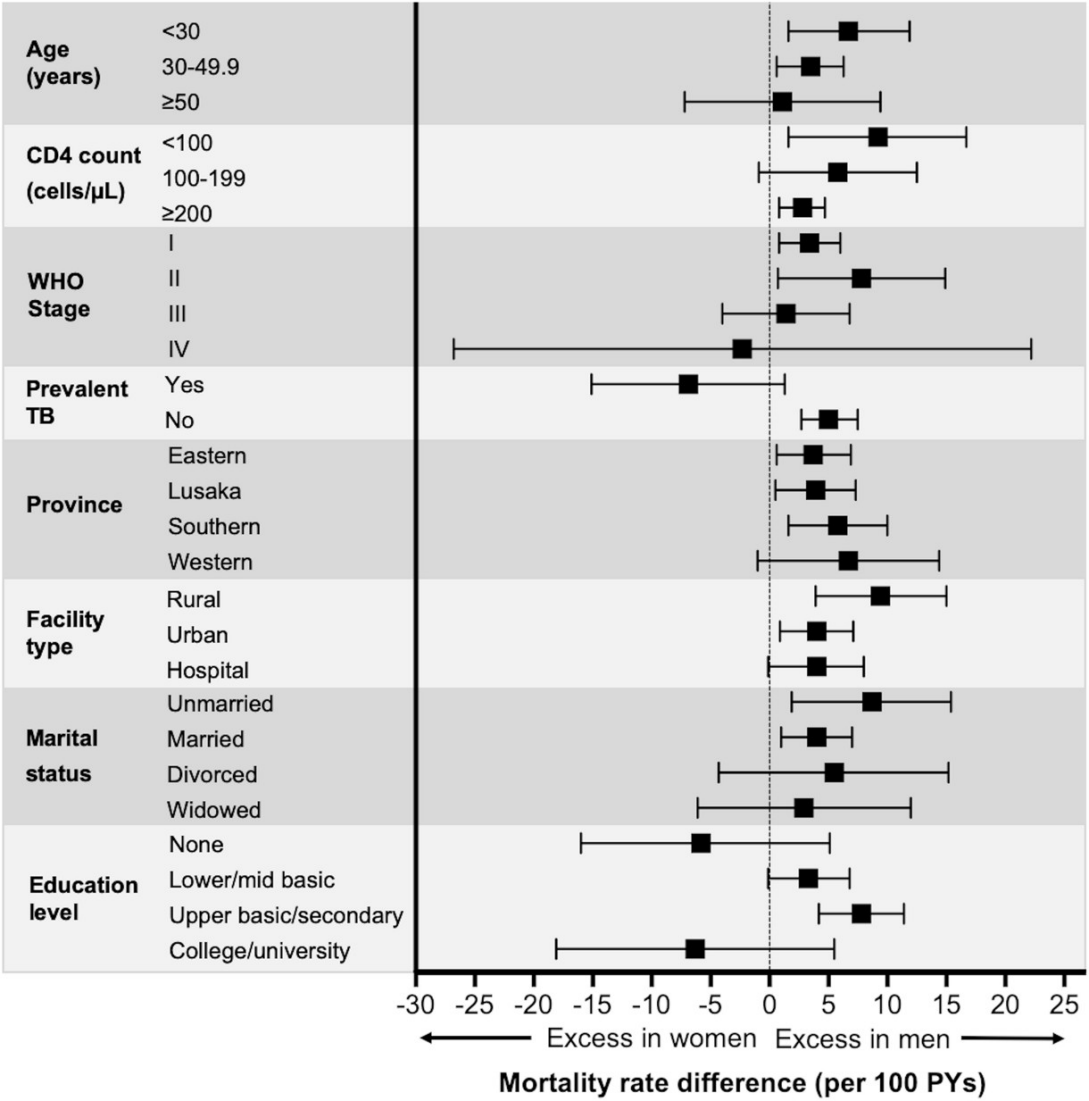
Forest plots demonstrating the difference in mortality rate (per 100 PYs) between men and women by characteristics at the time of ART initiation. Squares represent the difference in mortality rates while bars correspond to the 95% confidence intervals. A confidence interval crossing 0 (dotted vertical line) suggests that there is no evidence of a difference in sex-specific mortality rates, while a value greater than 0 suggests an excess of mortality among men and a value less than 0 suggests an excess of mortality among women. PYs, person-years; TB, tuberculosis.

Overall, after incorporating the results of tracing, men had a mortality rate of 10.3 (95% CI 8.2–12.4) deaths/100 person-years (PYs), compared to 5.5 (95% CI 4.3–6.8) deaths/100 PYs among women (difference +4.7 [95% CI 2.3–7.2]/100 PYs; rate ratio 1.9 [95% CI 1.3–2.4]; *p <* 0.001). Large disparities in mortality rates between men and women were observed in most subgroups, with excess mortality consistently concentrated among men (S3 Table; Fig 1). We found especially large differences in mortality rates between men and women in those who were <30 years old (+6.7 deaths/100 PYs difference; *p* = 0.010), had initial CD4 count < 100 cells/μl (+9.2 deaths/100 PYs difference; *p* = 0.017), had WHO stage II disease at ART initiation (+7.7 deaths/100 PYs difference; *p* = 0.033), attended rural health centers (+9.4 deaths/100 PYs difference; *p <* 0.001), were unmarried (+8.7 deaths/100 PYs difference; *p* = 0.014), and had some secondary education (+8.7 deaths/100 PYs difference; *p* < 0.001). Not surprisingly, the highest absolute mortality rates were observed among those with CD4 count < 100 cells/μl (men: 19.2 [95% CI 12.5–25.8] deaths/100 PYs) and those with WHO stage IV disease at ART initiation (men: 24.6 [95% CI 7.8–41.3] deaths/100 PYs; women: 26.9 [95% CI 9.0–44.7] deaths/100 PYs) (S3 Table; Fig 1).

### Mortality rates among men and women by health facility type

**Fig 2:**
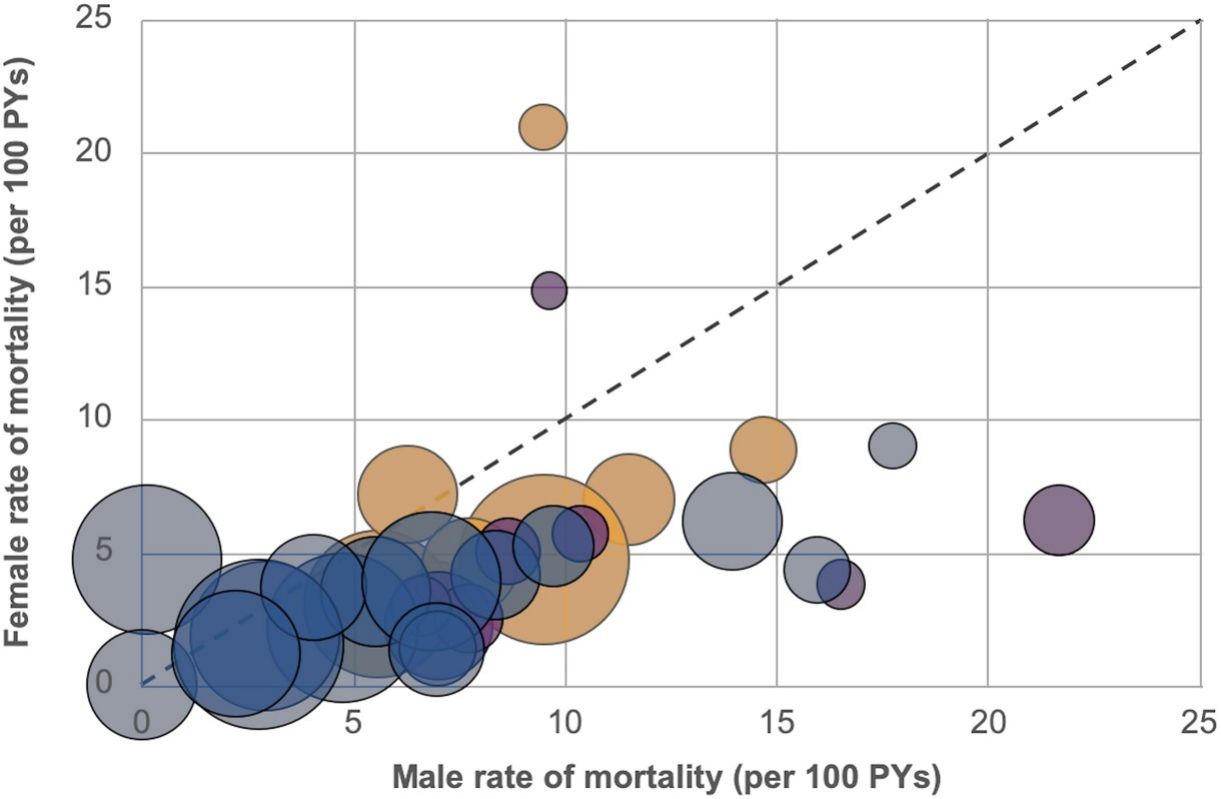
Bubble plot of the mortality rate (in person-years [PYs]) among men and women receiving antiretroviral therapy in Zambia. The bubbles represent 32 health facilities and are sized proportionally to the number of patients each clinic contributes to the overall analysis. The color of the bubbles denotes the facility type: blue = urban, purple = rural, and orange = hospital. The dashed line represents sex parity with respect to mortality rates; for example, any bubble to the right of the dashed line represents excess mortality among men, while a bubble to the left of this line represents excess mortality among women.

Next, we sought to evaluate for the presence of heterogeneity in mortality rates among men and women attending individual health facilities in an urban, rural, or hospital setting. Fig 2 shows a bubble plot of sex-stratified mortality rates by clinic. Men had higher mortality rates than women at 28 of 32 clinics. In addition, we found larger facility-to-facility differences among men (IQR 5.5–9.9 deaths/100 PYs), whereas mortality among women was far more similar across facilities (IQR 2.4–5.8 deaths/100 PYs). Men attending rural or hospital facilities appeared to have higher mortality rates than those attending urban facilities; however, this difference did not reach statistical significance.

### Mortality rates among men and women by time on ART

To evaluate whether differential mortality between men and women was present throughout the first 2 years of ART, we calculated sex-specific mortality rates according to the duration of time on ART (Table 2).

**Table 2:** Mortality rates (per 100 person-years) among men and women stratified according to duration of ART.

Duration on ART	Mortality rate or rate difference (95% CI)		
	Rate among men	Rate among women	Rate difference
<6 months	10.4 (7.8 to 12.9)	5.7 (4.1 to 7.2)	4.7 (1.7 to 7.7)
6–11.9 months	7.3 (4.3 to 10.4)	3.3 (1.4 to 5.2)	4.0 (0.4 to 7.6)
12–24 months	14.1 (7.0 to 21.1)	8.7 (4.7 to 12.7)	5.4 (−2.7 to 13.5)

High mortality rates were seen among men in the first 6 months of ART, and the sex-specific mortality rates were largest during this time (difference +4.7 [95% CI 1.7–7.7]/100 PYs; *p <* 0.001). While mortality rates appeared to decline for both men and women at 6–11.9 months of ART, excess mortality was still observed among men. After 1 year of ART, men and women experienced increased rates of mortality compared to the preceding 6 months, but mortality did not differ substantially by sex.

### Predicted mortality rates among men and women by age

**Fig 3:**
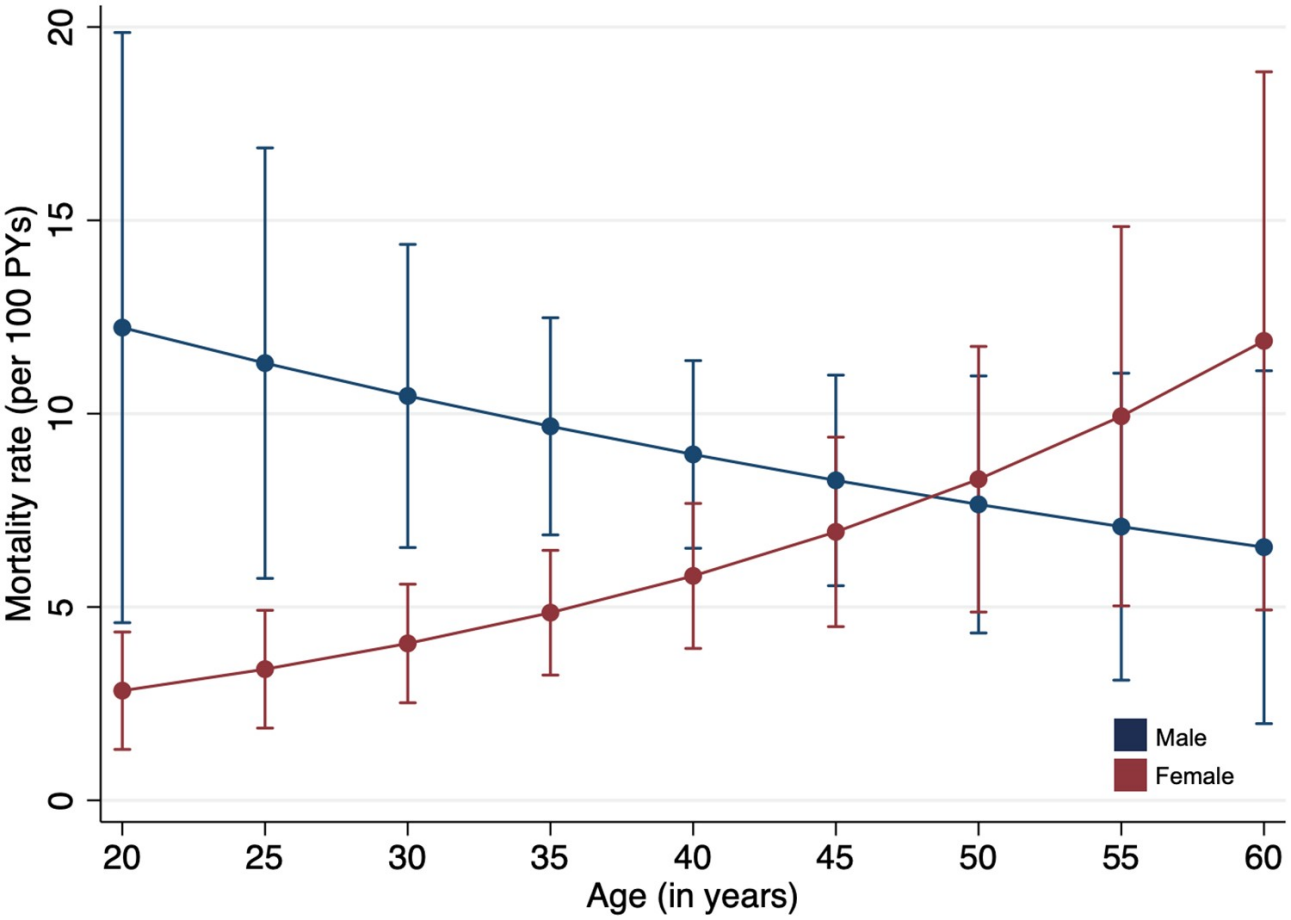
Plot of the predicted mortality rates among men and women according to age category. A multivariable Poisson regression model was adjusted for CD4 count (coded as a continuous variable), year of ART initiation, relationship status, education status, and province; there was evidence of effect modification between age (coded as a variable) and sex, and this interaction term was included in the model. The circles represent the predicted mortality rate at a defined age, and the bars correspond to the 95% confidence intervals. PYs, person-years.

Next, we determined predicted mortality rates among men and women according to age (Fig 3). After adjusting for other potential confounding and mediating variables, the largest differences in mortality rates remained between men and women <30 years old. While the highest absolute mortality rates among men were among those <30 years old, mortality did not significantly differ by age. However, among women, there was a clear pattern of increasing mortality rates among women with age—this was exemplified by the finding that women ≥50 years old had substantially higher predicted mortality rates than women <30 years old, but similar mortality rates compared to men of the same age.

### Care engagement status among men and women during ART

**Fig 4:**
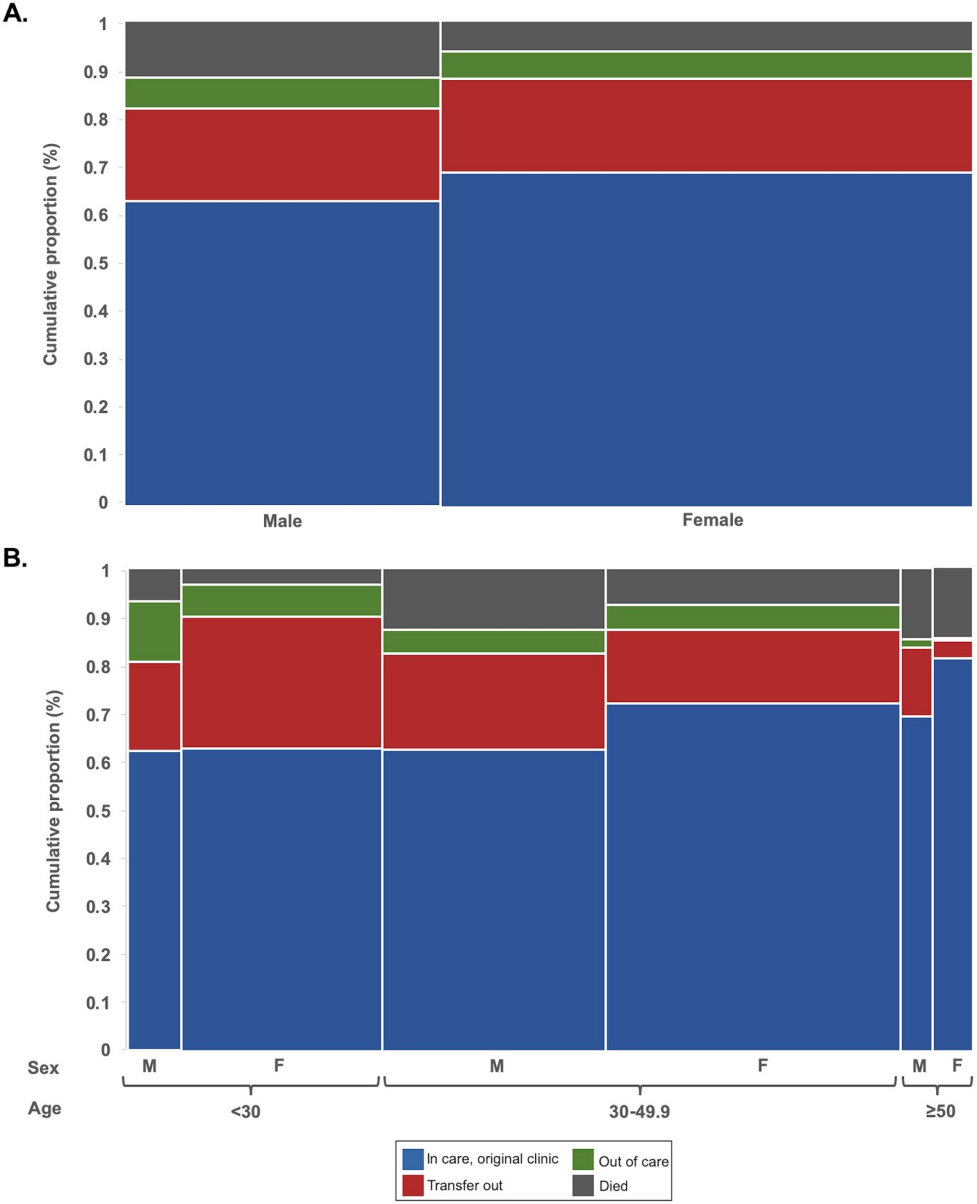
Mosaic plot demonstrating HIV care engagement status in the first 18 months of ART (*n* = 49,129) overall by sex, and by sex and age category. (A) By sex; (B) by sex and age category. Patients were categorized into 1 of 4 mutually exclusive categories: (1) alive and in care at original clinic, (2) alive and in care after transferring to a new clinic, (3) alive but out of care, or (4) dead. F, female; M, male.

Estimates of care engagement status among men and women during the first 18 months of ART that incorporate the results of intensive tracing are presented in Fig 4A. Among all men by 18 months, 11.8% (95% CI 11.1%–12.5%) had died, 6.2% (95% CI 5.7%–6.7%) were alive but out of care, 19.1% (95% CI 18.2%–20.1%) had transferred to a new clinic, and 62.9% (95% CI 60.7%–65.0%) remained in care at their original clinic. In direct comparison, 6.4% (95% CI 6.0%–6.8%) of women had died, 5.6% (95% CI 5.2%–6.0%) were alive but out of care, 19.3% (95% CI 18.6%–20.0%) had transferred to a new clinic, and 68.7% (95% CI 67.2%–70.2%) remained in care at their original clinic. Thus, men had a 5.4% higher cumulative proportion of mortality during the first 18 months after ART initiation, but little difference was otherwise observed between men and women with respect to the cumulative proportion of those who transferred or disengaged from care.

### Care engagement status among men and women during ART by age

To further evaluate differential retention in HIV services between men and women during ART, cumulative estimates of care engagement status during the first 18 months of ART were determined, stratified according to age group (Fig 4B). Compared to women of the same age, men <30 years were more likely to be disengaged from care (13.3% [95% CI 11.5%–15.3%] versus 6.7% [95% CI 6.1%–7.4%]) or to have died (7.1% [95% CI 6.1%–8.2%] versus 3.4% [95% CI 3.0%–3.9%]). Among individuals 30–49.9 years old, men again had a higher cumulative mortality (12.8% [95% CI 11.9%–13.7%] versus 7.7% [95% CI 7.1%–8.4%]); however, the probability of being alive and disengaged from ART services did not differ between men and women. Extremely high rates of mortality were seen among both men and women ≥50 years old (Fig 4B): 14.8% (95% CI 12.4%–17.4%) of men and 14.6% (95% CI 12.3%–17.2%) of women died during the first 18 months of ART. Notably, only 1.7% (95% CI 0%–2.6%) and 0.6% (95% CI 0%–1.2%) of men and women ≥50 years old, respectively, were estimated to be alive but out of care.

There was little difference with respect to men’s likelihood of transferring between ART clinics among different age groups (range: 14.4% [among those ≥50 years old] to 20.0% [among those aged 30–49.9 years old]); however, women <30 years old were more than 7 times more likely to transfer between ART clinics than those ≥50 years old (27.2% versus 3.5%).

## Discussion

A multistage sampling approach was used to combine large-scale data from patients who remained in care with those from patients who were previously LTFU—this allowed us to determine regionally representative estimates of mortality among adult men and women newly initiating ART in Zambia. Among 49,129 adults with HIV newly initiating ART in Zambia, we found large differences in survival between men and women, especially among individuals less than 30 years of age. We also found that older women, 50 years of age or older, were particularly at risk: They experienced a mortality rate similar to that of their male counterparts and had a substantially higher rate of mortality than women less than 30 years old—this has not previously been well appreciated.

Our study builds upon literature that has demonstrated differential mortality experienced by men with HIV [21,23,24,39,40]. The findings we present are also consistent with broader health and survival inequities experienced by men in most settings globally across all ages, both overall and for a number of specific health conditions [25,41,42]. However, to our knowledge, this is the first study to determine regionally representative age-stratified mortality estimates among men and women newly initiating ART at public health facilities by incorporating the outcomes of patients previously LTFU. Similar to other programs from sub-Saharan Africa [39,43], we found that a slightly higher proportion of men were LTFU. Our results concur with an individual patient data meta-analysis among 7,377 patients lost from ART services in sub-Saharan Africa and subsequently traced, which found that male sex was a strong predictor of death (adjusted hazard ratio = 1.6) [44].

Disparities in survival between men and women were most marked among those who were young and among those who were unmarried. After adjustment for potential confounders, men less than 30 years old were substantially more likely to die after ART initiation than women of the same age. Younger men who remained alive also had high rates of disengagement from ART services, putting them at high risk for HIV viremia and poor HIV-related outcomes [29]. Notably, large differences in mortality rates between men and women were apparent among those who were unmarried at the time of ART initiation. Unmarried men had an excess mortality rate of 8.7 deaths/100 PYs compared to unmarried women; nearly one-third of men less than 30 years old reported that they were unmarried. These findings highlight the crucial need to better understand the behaviors and preferences of men living with HIV [45–48], in order to identify improved and specific engagement and retention strategies—especially among those who are young and may lack social or partner support, as such individuals traditionally have poor engagement throughout the HIV care continuum [49,50]. While optimum strategies are not well known, these may include improved access to self-testing [51], use of incentives [8], and male-friendly services that emphasize confidentiality and include flexible hours, integrated care, and reduced visit frequency [52,53], as well as decentralization of services such as through community adherence groups [1,8,51,54]. Furthermore, effective retention support mechanisms—such as undertaking early and intensive tracing among patients LTFU from ART services (as soon as 1 week after loss to follow-up) via phone calls, text messages, or community visits—may be an important strategy towards improving HIV outcomes among men, but require further study [55–61]. The recently published SEARCH Study may provide clues as to how better to reach and retain men [62]. It enrolled more than 300,000 individuals in Kenya and Uganda and evaluated the impact of a community-based test-and-treat strategy as part of a multi-disease approach, coupled with streamlined ART delivery; after 3 years, the study intervention was associated with an elimination of differential mortality between men and women [63].

We also found large disparities in survival between men and women in several additional subgroups. These included men with CD4 count < 100 cells/μl, those with WHO stage II disease at initiation, and those without prevalent TB at ART initiation. Men attending clinics in rural settings, where they may have poor access to services, were also found to have disproportionately higher rates of mortality. Of note, heterogeneity was present with respect to sex-specific differences by health facility—while men were more likely to die than women at 90% of the primary health facilities evaluated across 4 provinces, men’s rates of mortality varied substantially across individual clinics, from 0 to 39.5 deaths per 100 PYs, and were up to 8.5 times higher than women’s mortality rates at the same clinic. These data may in part reflect different facility-specific attitudes towards and proficiency in reaching and retaining men in ART services. These differences highlight the need not only to improve engagement and retention among men in these settings, but also to improve identification and timely referral mechanisms for sick men and women to the appropriate level of care service (e.g., more frequent outpatient monitoring and/or visits or linkage to referral hospitals), especially in more remote settings.

The reasons underpinning our finding of a large male survival deficit among adults with HIV in Zambia (and other settings in sub-Saharan Africa) are not entirely clear. The most commonly cited explanation is differential and late engagement in HIV services [23,24]; consistent with prior reports of delayed health-seeking among men living with HIV in sub-Saharan Africa [4], men in our study who presented to HIV care tended to be older, to have a greater degree of immunodeficiency, and to have more advanced disease at the time of ART initiation than women. The possibility of delayed health-seeking by men is further supported by our finding that the highest absolute mortality rates among men, as well as the largest differential mortality rates compared to women, occurred among those with initial CD4 count < 100 cells/μl and during the first 6 months of ART. Men less than 30 years also had high rates of program disengagement, although among older patients, retention patterns did not significantly differ between men and women. Additionally, men have often been found to have poorer adherence to ART [2,6,64] and may, therefore, not fully benefit from the life-saving benefits of ART even if they are “retained in care.” While not reported as part of this study, among this same population, we have previously found male sex to be associated with viremia after ART initiation [29]. It is notable, however, that prior studies have found persistently elevated mortality among men during ART that are not entirely explained by baseline differences in HIV disease status, or differences in virologic suppression, CD4 count recovery, or rates of loss to follow-up [24]. In the present study, we observed an excess mortality among men after adjusting for several baseline characteristics, including CD4 cell count, that was concentrated among younger men. Collectively, these findings suggest that, at least in part, the mortality excess noted among men in sub-Saharan Africa is likely attributable to causes independent of HIV [24,65].

TB remains the leading cause of death among people living with HIV [66], and it is not well appreciated that men are on average 2 times more likely to have prevalent TB disease than women, have lower engagement throughout the TB care cascade, and are more likely to die from TB [67–69]. Because routine program data were used, we were unable to ascertain the causes of death to identify factors that may be contributing to survival differences among men and women. However, 1 study based in Kwazulu-Natal, South Africa, investigated cause-specific mortality after finding that HIV-positive men lived on average 11.1 years less than HIV-positive women. Utilizing the demographic surveillance system, they found that TB accounted for 9 years (78.6%) of the sex-specific life-year difference, while injuries accounted for 1.7 years (15.7%) of the difference. While we did not systematically investigate for TB prior to or during ART, higher mortality rates were seen among men without known prevalent TB at ART initiation. These data collectively suggest that TB may be an important cause of death among men living with HIV in Zambia and point to the need for earlier and improved TB case detection among men and expanded coverage of isoniazid preventive therapy as possible strategies for reducing the male survival deficit.

Older women, ≥50 years of age, were found to be at extremely high risk for mortality. Not only were such women much more likely to die than younger women, less than 30 years old, but mortality rates were comparable to those of men ≥50 years old. More than 15% of those ≥50 years of age died within 18 months of initiating ART. As HIV-infected adults age, they are at increased risk for age-related co-morbidities such as hypertension, diabetes, and cardiovascular diseases [34]; however, this alone is unlikely to account for these findings. Late presentation to care, with the associated more advanced disease, likely underpinned some of the high mortality rates among older women, evidenced by older women being significantly more likely to have CD4 count < 200 cells/μl as well as WHO stage III/IV disease at ART initiation compared to younger women. There is also some evidence suggesting that older individuals may not experience immune recovery as quickly as younger individuals, which may prolong their time at risk [70]. Because mortality rates did not differ between men and women ≥50 years, one possible explanation for why younger women (those of reproductive age) may seemingly do better in HIV care than men is women’s engagement in reproductive health services, which may facilitate earlier diagnosis and linkage to care. Thus, our findings may suggest in part that sex-specific mortality differences do not reflect inherently better health behaviors among women compared to men, but rather access to reproductive health services and earlier HIV care entry among younger women, and a lack of similar benefit among older women who do not access such services [4,13]. It is well-established that HIV-positive women are at increased risk for the development of cervical cancer [71]. Cervical cancer is the most commonly diagnosed cancer among women in Zambia, with the highest proportion of registered cases concentrated among those 40–60 years old [72]. While data regarding cervical cancer were not available as part of our study, it is possible that delayed and missed diagnoses of cervical cancer may have also contributed to the high mortality rate observed among older women, and, therefore, continued scale-up and availability of screening [71] coupled with early ART and retention in HIV services [73] might be an important intervention to address mortality among older HIV-positive women. We also observed that less than 30% of women ≥50 years old were married (with more than 30% reporting being widowed), a marked difference from the nearly two-thirds of men ≥50 years old reporting being married. Compared to men of the same age, older women are at higher risk for stigma, social isolation, and economic insecurity, especially after their husband’s death [74,75]. Further dedicated research is needed to understand the causes underpinning older women’s high rates of mortality, as well as novel interventions aimed at improving their individual outcomes.

Strengths of this study include the use of a novel sampling strategy to generate provincially representative estimates of HIV care status among nearly 50,000 adults living with HIV initiating ART at public facilities in Zambia. There were, however, some limitations. This study utilized routine programmatic data obtained from national electronic medical records that were not maintained by research staff; therefore, such data may be incomplete or a very small proportion may be entered incorrectly (although this is a living medical record, and errors are rectified as new results are populated). However, for key explanatory variables (age and sex), the data were complete and are highly unlikely to be misclassified—we have previously found good reliability between paper charts and electronic entries. Sampling among those LTFU was performed randomly, but 26% of patients sampled could not be traced despite intensive efforts; such patients had similar baseline characteristics compared to those successfully traced, but if they had differential outcomes, then our results may be biased. However, we applied non-response weights to account for patients whose outcomes couldn’t be traced. While country-wide standardized mortality rates in Zambia are not available, we found that men aged 30 years old had an approximately 5 times higher mortality and women aged 50 years old had an approximately 3 times higher mortality compared to men and women of the same age in a prior population-based survey in Lusaka Province [76]; this suggests that mortality rates among young men and older women living with HIV in Zambia are substantially higher than would be expected in the general population. Additionally, our adjusted analyses included both baseline CD4 count and WHO stage at baseline, which may be on the causal pathway to mortality and may therefore be mediators rather than confounders. The estimates from these analyses therefore represent the direct effect of sex on mortality estimates across age; however, these estimates did not differ substantially from the total effect of sex on mortality when CD4 count and WHO stage were excluded from the adjusted analyses. Finally, these estimates reflect care engagement status among men and women between 2013 and 2015. Since this time, HIV services have been further decentralized in Zambia, and community adherence groups have been implemented in many settings [77,78]; it will be important to evaluate whether this model of care improves long-term retention among all adults living with HIV and whether the changes affect sex-specific differences in mortality.

In conclusion, by deriving unbiased estimates of mortality, we found large disparities in survival between men and women initiating ART in Zambia, with mortality highest among young men. Importantly, we also found high mortality rates among older women that have not previously been well described. This analysis demonstrates the need for not only continued novel and sustained efforts to improve male engagement throughout the HIV care continuum, but also tailored interventions targeting young men and older women who may be at the highest risk of poor HIV-related outcomes.

## Supporting information

S1 Appendix Study protocol. (PDF)

S1 STROBE Checklist STROBE statement checklist. (DOCX)

S1 Fig Flowchart showing overview of study weighting strategy. (TIF)

S2 Fig Flowchart showing study overview including sampling strategy and patient outcomes. ART, antiretroviral therapy; CIDRZ, Centre for Infectious Disease Research in Zambia; IPW, inverse probability weight; LTFU, lost to follow-up.
(TIF)

S1 Table Overview of patients lost to follow-up and tracing outcome status stratified by sex and age among patients newly initiating ART in Zambia. (DOCX)

S2 Table Baseline characteristics of patients lost to follow-up who were selected for tracing. (DOCX)

S3 Table Sex-stratified mortality rates and rate differences (per 100 person-years) among adults with HIV newly initiating ART in Zambia. (DOCX)

## Abbreviations

ART: antiretroviral therapy
LTFU: lost to follow-up
PYs: person-years
TB: tuberculosis
