# Supplementary material for: Mortality estimates by age and sex among persons living with HIV after ART initiation in Zambia using electronic medical records supplemented with tracing a sample of lost patients: A cohort study
Source: PLoS Med. 2020 May 13;17(5):e1003107. doi: 10.1371/journal.pmed.1003107 (PMC7219718; doi:10.1371/journal.pmed.1003107)
Supplement: S1 STROBE Checklist — (DOCX) [file pmed.1003107.s002.docx]

STROBE Statement—Checklist of items that should be included in reports of cohort studies

|  | **Item No** | **Recommendation** | **Response:** |
| --- | --- | --- | --- |
| **Title and abstract** | | | |
|  | 1 | a) Indicate the study’s design with a commonly used term in the title or the abstract | The design is included in the study title - "population based estimates from an electronic medical Population-based estimates using an electronic medical records-based cohort supplemented with tracing a sample of patients with unknown outcomes.” |
|  |  | b) Provide in the abstract an informative and balanced summary of what was done and what was found | This is provided (***see abstract).*** |
| **Introduction** | | | |
| Background | 2 | Explain the scientific background and rationale for the investigation being reported | This is described in the background section (see ***Background section paragraphs 2 and 3).*** |
| Objectives | 3 | State specific objectives, including any prespecified hypotheses | Specific objectives are stated in the background section (see ***Background section paragraph 3).*** |
| **Methods** | | | |
| Study design | 4 | Present key elements of study design early in the paper | This is provided (***see Methods Section, setting and patient sampling sub-section, paragraph 1).*** |
| Setting | 5 | Describe the setting, locations, and relevant dates, including periods of recruitment, exposure, follow-up and data collection | This is provided (***see Methods Section, setting and patient sampling sub-section, paragraph 1).*** |
| Participants | 6 | a) Give the eligibility criteria, and the sources and methods of selection of participants. Describe methods of follow-up | This is provided (***see Methods Section, setting and patient sampling sub-section, paragraph 1).*** |
|  |  | b) For matched studies, give matching criteria and number of exposed and unexposed | Not applicable |
| Variables | 7 | Clearly define all outcomes, exposures, predictors, potential confounders, and effect modifiers. Give diagnostic criteria if applicable | Outcomes, potential confounders and effect modifiers are described in detail (***see Methods Section, analysis sub-section, paragraph 1).*** Confounders and effect modifiers are also described in the Figure 3 legend. |
| Data sources/ measurements | 8 | For each variable of interest, give sources of data and details of methods of assessment. Describe comparability of assessment methods if there is more than one group | All data sources and methods of obtainment for variables of interest are described in detail (***see Methods Section, procedures and measurements sub-section, paragraph),*** |
| Bias | 9 | Describe any efforts to address potential sources of bias | There are a few potential sources of bias that we discuss. One is the use of routine medical records, which may be incompletely documented. A second is that approximately one quarter of LTFU patients sampled could not be traced; we show that baseline demographics do not differ between those traced and those who could not be traced (***see S3 Table).*** We discuss both of these as potential sources of bias in the strengths and limitations section of the discussion section (***See Discussion – paragraph 8).*** *We also carefully address the problem of missingness,* ***see item 14b below****.* |
| Study size | 10 | Explain how the study size was arrived at | We have detailed in the manuscript our sampling strategy for the entire cohort and those who were included in the present gender-specific analysis among those newly starting ART (***see Methods Section, setting and patient sampling sub-section, paragraph 1, S2 Figure and also S1 appendix*).** We have also provided an overview of the weighting strategy used in the study (***see S1 Figure*).** |
| Quantitative variables | 11 | Explain how quantitative variables were handled in the analyses. If applicable, describe which groupings were chosen and why | For all analyses, we have made it clear whether quantitative analyses were categorized according to recognized thresholds or were included as a continuous variable (***see Methods Section, analysis sub-section, paragraph 1 and Figure 3 legend).*** |
| Statistical methods | 12 | (a ) Describe all statistical methods, including those used to control for confounding | These details are provided in the methods section (***see Methods Section, analysis sub-section, paragraph 1).*** |
|  |  | (b ) Describe any methods used to examine subgroups and interactions | These details are provided in the methods section (***see Methods Section, analysis sub-section, paragraph 1 and Figure 3 legend).*** |
|  |  | (c) Explain how missing data were addressed | Sources of missing data and our approach to addressing missing data are described in detail (***see Methods Section, analysis sub-section, paragraph 1).*** |
|  |  | (d ) If applicable, explain how loss to follow-up was addressed | Not applicable; tracing patients lost-to-follow-up was central to the study methodology and the approach is described in detail. |
|  |  | (e ) Describe any sensitivity analyses | No sensitivity analyses were conducted. |
| **Results** |  |  |  |
| Participants | 13 | (a) Report numbers of individuals at each stage of study—eg numbers potentially eligible examined for eligibility, confirmed eligible, included in the study, completing follow-up, and analysed | This information is described in results section and is also summarized in a flow chart (***See Results section, paragraph 1 and Figure S2).*** |
|  |  | (b) Give reasons for non-participation at each stage | This information is described in results section and is also summarized in a flow chart (***See Results section, paragraph 1 and Figure S2).*** |
|  |  | (c) Consider use of a flow diagram | A flow chart is provided in the results section (***See Results section, Figure S2).*** |
| Descriptive Data | 14 | (a) Give characteristics of study participants (eg demographic, clinical, social) and information on exposures and potential confounders | This information is provided in the results section (***See Results section, paragraphs 1 and 2, and Table 1).*** |
|  |  | (b) Indicate number of participants with missing data for each variable of interest | This information is provided in Table 1 of the results section by noting an “unknown” classification for each variable where relevant (***See Results section, Table 1).*** |
|  |  | (c) Summarise follow-up time (eg, average and total amount) | All efforts to trace lost patients were done between Oct 2015 and June 2016 as described in the methods section and follow-up time was accrued was between August 1, 2013 and July 31, 2015 as described in both the methods section and the results section (***see Methods Section, Setting sub-section, paragraph 1, Procedures sub-section, paragraph 1 and Results section, paragraph 1).*** |
| Outcome data | 15 | Report numbers of outcome events or summary measures over time | Revised mortality rates and the proportion of patients in each care state over time are reported in the results section (***see Results section, Table 2, Figures 1-4, and S4 Table).*** |
| Main Results | 16 | (a) Give unadjusted estimates and, if applicable, confounder-adjusted estimates and their precision (eg, 95% confidence interval). Make clear which confounders were adjusted for and why they were included | This information has been provided as described in item 15 and adjusted analyses, including confounders/interaction terms are shown and described in addition to unadjusted estimates (***see Results Section, Predicted mortality rates sub-section, Figure 3)*** |
|  |  | (b) Report category boundaries when continuous variables were categorized | These are described in the results section (***see Results Section, Tables 1 and 2, S2 and S3 Tables)***. However, for adjusted, multivariable analyses, continuous variables (age and CD4 count) were included in the model as continuous variables (***see Results Section, Figure 3, figure legend 3).*** |
|  |  | (c ) If relevant, consider translating estimates of relative risk into absolute risk for a meaningful time period | We have provided mortality estimates by sex as absolute rates and rate differences (***see Results Section, Tables 2, S3 Table, Figures 1 and 2).*** We have also provided estimates of the cumulative proportion of patients in each clinical care state in the first 18 months of ART *(****see Results Section, Figure 4).*** |
| Other analyses | 17 | Report other analyses done—eg analyses of subgroups and interactions, and sensitivity analyses | We present all analyses in the paper with additional analysis methods in the supplementary materials. There were no additional analyses not presented in the full manuscript. |
| **Discussion** |  |  |  |
| Key Results | 18 | Summarise key results with reference to study objectives | Our discussion section summarizes key results with reference to the study objectives defined in the final paragraph of the background section (***see Discussion Section)*** |
|  | 19 | Discuss limitations of the study, taking into account sources of potential bias or imprecision. Discuss both direction and magnitude of any potential bias | We provide a discussion on limitations and potential sources of bias as well as the potential implications of such bias *(****see Discussion Section, paragraph 8).*** |
| Interpretation | 20 | Give a cautious overall interpretation of results considering objectives, limitations, multiplicity of analyses, results from similar studies, and other relevant evidence | We have attempted to provide a conservative interpretation of our study results in the Discussion section making clear whenever possible what represents a hypothesis and what represents actual study findings *(****see Discussion Section)*** |
| Generalisability | 21 | Discuss the generalisability (external validity) of the study results | This is described *(****see Discussion Section, paragraph 8).*** |
| **Other information** |  |  |  |
| Funding | 22 | Give the source of funding and the role of the funders for the present study and, if applicable, for the original study on which the present article is based | This is described (***see section on Funding below authors information).*** |
