# Supplementary figures and images for: Mortality estimates by age and sex among persons living with HIV after ART initiation in Zambia using electronic medical records supplemented with tracing a sample of lost patients: A cohort study
Source: PLoS Med. 2020 May 13;17(5):e1003107. doi: 10.1371/journal.pmed.1003107 (PMC7219718; doi:10.1371/journal.pmed.1003107)

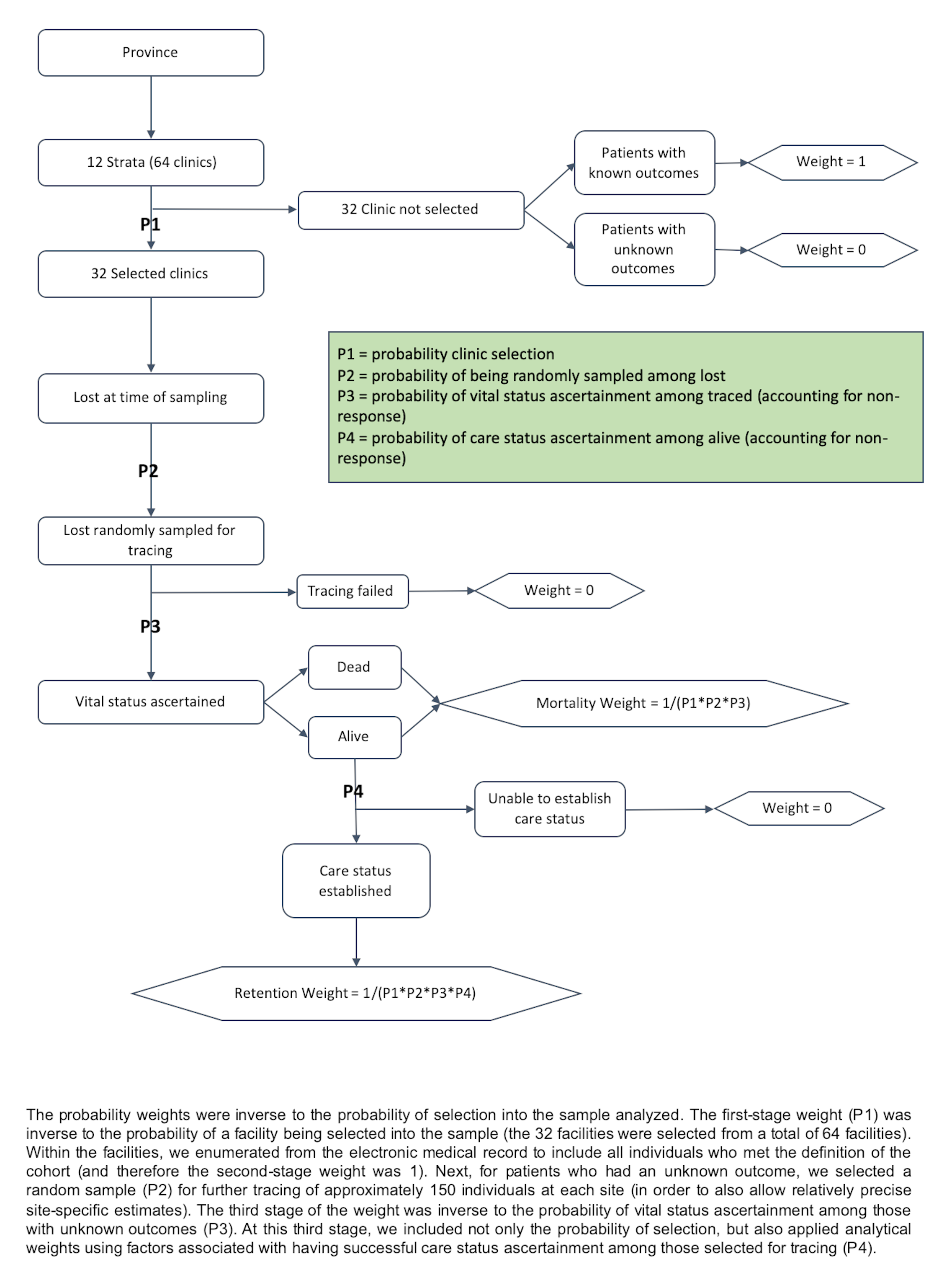

Supplement: S1 Fig — (TIF) [file pmed.1003107.s003.tif]

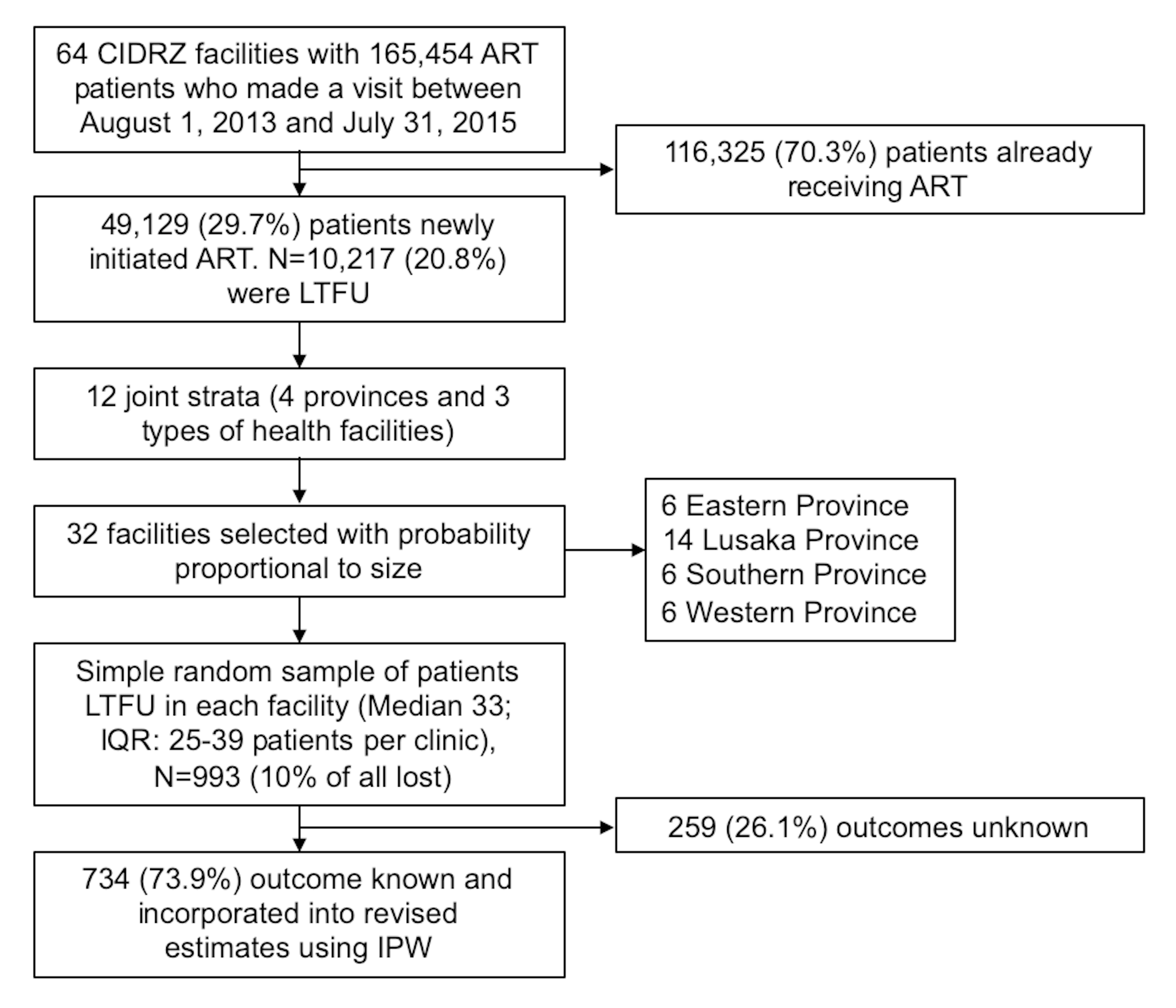

Supplement: S2 Fig — ART, antiretroviral therapy; CIDRZ, Centre for Infectious Disease Research in Zambia; IPW, inverse probability weight; LTFU, lost to follow-up. (TIF) [file pmed.1003107.s004.tif]
