# Supplementary material for: Mortality estimates by age and sex among persons living with HIV after ART initiation in Zambia using electronic medical records supplemented with tracing a sample of lost patients: A cohort study
Source: PLoS Med. 2020 May 13;17(5):e1003107. doi: 10.1371/journal.pmed.1003107 (PMC7219718; doi:10.1371/journal.pmed.1003107)
Supplement: S1 Table — (DOCX) [file pmed.1003107.s005.docx]

|  | **Male,**  **Overall** | **Female, Overall** |  | **Male,**  **<30** | **Female, <30** | **Male,**  **30-49.9** | **Female,**  **30-49.9** | **Male,**  **≥50** | **Female, ≥50** |
| --- | --- | --- | --- | --- | --- | --- | --- | --- | --- |
| **Total number starting ART** | 18,271 | 30,858 |  | 3,209 | 11,555 | 12,969 | 16,971 | 2,079 | 2,311 |
| **LTFU at the time of tracing, n (% of total starting ART)** | 3,981 (21.8) | 6,236 (20.2) |  | 803 (25.0) | 2,843 (24.6) | 2,779 (21.4) | 3,033 (17.9) | 393 (18.9) | 354 (15.3) |
| **Tracing undertaken, n (% of total LTFU)** | 397 (10.0) | 596 (9.5) |  | 77 (9.6) | 246 (8.7) | 281 (10.1) | 309 (10.2) | 38 (9.7) | 39 (11.0) |
| **Tracing successful, updated vital status obtained, n (% those selected for tracing)** | 306 (77.1) | 428 (71.8) |  | 56 (72.7) | 174 (70.7) | 219 (77.9) | 224 (72.5) | 30 (79.0) | 28 (71.8) |

**Supplementary Table 1**. Overview of patients lost-to-follow-up and tracing outcomes status stratified by sex and age among patients newly initiating antiretroviral therapy in Zambia (n=49,129)
