# Supplementary material for: Mortality estimates by age and sex among persons living with HIV after ART initiation in Zambia using electronic medical records supplemented with tracing a sample of lost patients: A cohort study
Source: PLoS Med. 2020 May 13;17(5):e1003107. doi: 10.1371/journal.pmed.1003107 (PMC7219718; doi:10.1371/journal.pmed.1003107)
Supplement: S2 Table — (DOCX) [file pmed.1003107.s006.docx]

**Supplementary Table 2. Baseline characteristics among patients lost-to-follow-up**

**who were selected for tracing**

|  | **Overall**  **(n=993)** | **Successfully traced**  **(n=734)** | **Could not be traced**  **(n=259)** |
| --- | --- | --- | --- |
| **Age at ART initiation (years), median (IQR)** | 32 (27-39) | 33 (27-40) | 31 (25-38) |
| **Male Gender** | 409 (41.2) | 311 (42.4) | 98 (37.8) |
| **Initiation CD4, median (IQR)** | 268 (121-423) | 256 (119-412) | 282 (127-444) |
| **WHO Stage at enrollment** |  |  |  |
| Stage 1 | 433 (43.6) | 321 (43.7) | 112 (43.2) |
| Stage 2 | 174 (17.5) | 130 (17.7) | 44 (17.0) |
| Stage 3 | 180 (18.1) | 138 (18.8) | 42 (16.2) |
| Stage 4 | 32 (3.22) | 22 (3.0) | 10 (3.9) |
| Unknown | 174 (17.5) | 123 (16.8) | 51 (19.7) |
| **Province** |  |  |  |
| Eastern | 170 (17.1) | 136 (18.5) | 34 (13.1) |
| Lusaka | 444 (44.7) | 305 (41.6) | 139 (53.7) |
| Southern | 196 (19.7) | 151 (20.6) | 45 (17.4) |
| Western | 183 (18.4) | 142 (19.4) | 41 (15.8) |
| **Facility type** |  |  |  |
| Rural | 227 (22.9) | 189 (25.8) | 38 (14.7) |
| Urban | 540 (54.4) | 386 (52.6) | 154 (59.5) |
| Hospital | 226 (22.8) | 159 (21.7) | 67 (25.9) |
| **Marital status** |  |  |  |
| Unmarried | 135 (13.6) | 108 (14.7) | 27 (10.4) |
| Married | 514 (51.8) | 514 (52.6) | 128 (49.4) |
| Divorced | 93 (9.4) | 65 (8.9) | 28 (10.8) |
| Widowed | 64 (6.5) | 46 (6.3) | 18 (7.0) |
| Unknown | 187 (18.8) | 129 (17.6) | 58 (22.4) |
