# Supplementary material for: Mortality estimates by age and sex among persons living with HIV after ART initiation in Zambia using electronic medical records supplemented with tracing a sample of lost patients: A cohort study
Source: PLoS Med. 2020 May 13;17(5):e1003107. doi: 10.1371/journal.pmed.1003107 (PMC7219718; doi:10.1371/journal.pmed.1003107)
Supplement: S3 Table — (DOCX) [file pmed.1003107.s007.docx]

**Supplementary Table 3.** Sex stratified mortality rates and rate differences (per 100 person-years) among adults with HIV newly initiating ART in Zambia.

|  |  | **Rate of Mortality, per 100 PYs (95%CI)** | | **Rate difference***  **(95%CI)** |
| --- | --- | --- | --- | --- |
|  |  | **Male** | **Female** |  |
| **Overall** |  | 10.3 (8.2-12.4) | 5.5 (4.3-6.8) | 4.7 (2.3-7.2) |
| **Age at study entry (years)** |  |  |  |  |
| 18-30 |  | 9.7 (4.7-14.6) | 2.9 (1.5-4.3) | 6.7 (1.6-11.9) |
| 30-49.9 |  | 9.6 (7.4.11.9) | 6.1 (4.4-7.8) | 3.5 (0.6-6.3) |
| ≥50 |  | 13.1 (6.9-19.3) | 12.0 (6.5-17.5) | 1.1 (-7.2-9.4) |
| **CD4 cell count (cells/uL)** |  |  |  |  |
| <100 |  | 19.2 (12.5-25.8) | 10.0 (6.4-13.6) | 9.2 (1.6-16.7) |
| 100-199 |  | 12.5 (7.0-18.0) | 6.7 (2.9-10.5) | 5.8 (-0.9-12.5) |
| ≥200 |  | 4.7 (2.9-6.5) | 1.9 (1.0-2.8) | 2.8 (0.8-4.7) |
| **WHO Stage at baseline** |  |  |  |  |
| I |  | 6.3 (4.0-8.6) | 2.9 (1.6-4.2) | 3.4 (0.8-6.0) |
| II |  | 15.4 (9.2-21.5) | 7.6 (4.0-11.2) | 7.7 (0.6-14.8) |
| III |  | 11.6 (7.4-15.8) | 10.3 (6.7-13.8) | 1.4 (-4.0-6.9) |
| IV |  | 24.6 (7.8-41.3) | 26.9 (9.0-44.7) | -2.3 (-26.8-22.2) |
| **TB status at baseline** |  |  |  |  |
| Known, prevalent TB |  | 4.7 (1.0-8.3) | 11.6 (4.2-18.9) | -6.9 (-15.1-1.3) |
| Unknown or negative for TB |  | 10.3 (8.3-12.4) | 5.3 (4.1-6.4) | 5.1 (2.7-7.5) |
| **Province** |  |  |  |  |
| Eastern |  | 9.3 (6.6-11.9) | 5.6 (3.8-7.3) | 3.7 (0.6-6.9) |
| Lusaka |  | 8.4 (5.5-11.3) | 4.5 (2.7-6.3) | 3.9 (0.5-7.3) |
| Southern |  | 12.4 (8.9-15.9) | 6.5 (4.3-8.8) | 5.8 (1.6-10.0) |
| Western |  | 14.0 (7.2-20.7) | 7.3 (3.6-10.9) | 6.7 (-1.0-14.4) |
| **Facility type** |  |  |  |  |
| Rural |  | 15.9 (10.9-20.9) | 6.5 (4.1-8.9) | 9.4 (3.9-15.0) |
| Urban |  | 8.6 (5.9-11.2) | 4.6 (3.0-6.2) | 4.0 (0.9-7.1) |
| Hospital |  | 10.5 (7.1-14.0) | 6.5 (4.4-8.7) | 4.0 (-0.1-8.0) |
| **Relationship status** |  |  |  |  |
| Unmarried |  | 13.3 (7.3-19.4) | 4.7 (1.6-7.7) | 8.7 (1.9-15.4) |
| Married |  | 8.6 (6.2-11.0) | 4.6 (2.9-6.4) | 4.0 (1.0-7.0) |
| Divorced |  | 14.4 (5.9-22.8) | 8.9 (4.0-13.7) | 5.5 (-4.3-15.2) |
| Widowed |  | 11.3 (3.1-19.6) | 8.4 (4.7-12.1) | 2.9 (-6.1-12.0) |
| **Education level** |  |  |  |  |
| None |  | 14.1 (5.0-23.2) | 9.0 (3.1-14.9) | 5.1 (-5.8-16.0) |
| Lower-mid basic |  | 8.9 (6.0-11.9) | 5.6 (3.8-7.4) | 3.3 (-0.1-6.8) |
| Upper basic/secondary |  | 11.4 (8.0-14.8) | 3.6 (2.3-4.9) | 7.8 (4.2-11.4) |
| College/University |  | 5.7 (3.3-8.1) | 12.0 (0.5-23.6) | -6.3 (-18.1-5.5) |

*Rate difference: a value greater than 0 suggests excess mortality among men compared to women, while a value less than zero suggests excess mortality among women compared to men. Abbreviations: TB=tuberculosis; WHO=World Health Organization.
